# Clinical correlates of perivascular adipose tissue in coronary artery disease and obesity

**DOI:** 10.3389/fphys.2025.1651249

**Published:** 2025-08-26

**Authors:** Sunil Upadhaya, Amitabh C. Pandey, Jose Wiley, Thierry H. Le Jemtel

**Affiliations:** Department of Medicine, Section of Cardiology, Tulane University School of Medicine, New Orleans, LA, United States

**Keywords:** epicardial adipose tissue, visceral abdominal fat, coronary artery disease, heart failure, perivascular adipose tissue

## Abstract

The adipose tissue surrounding the arterial and venous vasculature and microvasculature affects vascular reactivity and pathology, particularly when perivascular adipose tissue (PVAT) accumulates in overweight and obese states. In the absence of convenient techniques to measure local blood flow and adipose tissue volume, perivascular adipose tissue-related alterations are barely considered in clinical settings. Furthermore, perivascular adipose tissue accumulation frequently coexists with obesity, and obesity alone leads to functional and structural vascular alterations. The proximity of epicardial adipose tissue (EAT) to the coronary arteries provides a unique opportunity to study the effects of perivascular adipose tissue on vascular pathology and reactivity. As coronary atherosclerotic plaque inflammation contributes to the inflammatory response of the surrounding adipose tissue, pericoronary adipose tissue attenuation may predict the risk of acute coronary events. Finally, perivascular adipose tissue accumulation may mediate obesity-associated regional subclinical left ventricular dysfunction in the absence of coronary artery disease.

## Introduction

In the absence of convenient techniques to appraise the effects of perivascular adipose tissue (PVAT) on vascular health and reactivity, the interaction between the quantity and quality of PVAT and the vasculature has received little attention in clinical settings (Eugene Chen, 2018; Chatterjee et al., 2009; Agabiti-Rosei et al., 2018). However, the contiguity of epicardial adipose tissue (EAT) to the adventitia of coronary arteries in the atrioventricular and interventricular grooves provides a unique opportunity to examine the effects of the quantity and quality of PVAT on coronary vascular reactivity and pathology (Kim et al., 2020; Badimon et al., 2024). After a brief review of PVAT and EAT, we discuss whether pericoronary adipose tissue attenuation (PCATa) reliably reflects coronary atherosclerotic plaque inflammation and vulnerability and present correlative data that link EAT surrounding the left anterior descending (LAD) coronary artery to the reduced left ventricular (LV) global longitudinal strain (GLS) and, thereby, the subclinical regional impairment of LV systolic function in obesity.

### Perivascular adipose tissue

Variable amounts of adipose tissue (AT) surround most of the arterial vasculature and microvasculature, except in the brain and pulmonary vasculature (Guglielmi and Sbraccia, 2018). Three decades ago, Soltis and Cassis (1991) uncovered that PVAT modulates vascular reactivity in addition to vascular protection and thermogenesis. Healthy PVAT relaxes pre-contracted arteries in response to noradrenaline, angiotensin II, serotonin, or phenylephrine in healthy subjects (Soltis and Cassis, 1991; Agabiti-Rosei et al., 2018). PVAT modulates vascular reactivity through the release of molecules and chemicals that alter the vascular tone via paracrine and endocrine actions (Balakumar et al., 2024). Adipokines released by PVAT exert vasoactive effects on the coronary arteries (Kim et al., 2020; Chen et al., 2021). In lean states, adiponectin, omentin, and adipocyte-derived relaxing factor (ADRF) induce vasorelaxation through adenosine triphosphate (ATP)-dependent potassium (K) channels, voltage-gated K channels, and a nitric oxide (NO)-dependent mechanism on endothelial and vascular smooth muscle cells (VSMCs) (Huang Cao et al., 2017). Aging, obesity, and vascular injury promote inflammation, heightened oxidative stress, and increased production of vasoactive molecules and chemicals by PVAT (Costa et al., 2018; Chen et al., 2021). Inflamed PVAT promotes VSMC proliferation, thickens the arterial wall, and produces vasoconstricting molecules and chemicals that annihilate the anticontractile properties of healthy EAT (Huang Cao et al., 2017). Increased adipocyte area and inflammation lead to the loss of the PVAT dilator effect in patients with metabolic syndrome and obesity (Greenstein et al., 2009). Furthermore, obesity-associated immune cell infiltration of hypertrophic perivascular adipocytes, oxidative stress, and pro-inflammatory cytokines promote VSMC proliferation through the release of PVAT-derived factors and increased local uptake of norepinephrine (Fernandez-Alfonso et al., 2013; Ahmad et al., 2019).

Studying the structural and functional effects of obesity on PVAT is arduous for several reasons. The PVAT does not exclusively consist of white adipocytes. Perivascular adipocytes can be white, beige, or brown and exert a variety of vascular effects at different sites along the conduit vessels (Huang Cao et al., 2017). Generally, PVAT consists of white adipocytes in resistance vessels and brown and white adipocytes in large conduit vessels (Huang Cao et al., 2017). Adipocytes from ectopic AT depots, including perivascular adipocytes, are derived from distinct and specific embryonic lineages (Huang Cao et al., 2017). Nutrient intake–energy expenditure mismatch leads to the accumulation of lipids in the subcutaneous AT (SAT) (Gustafson and Smith, 2015). When excessive nutrient intake exceeds the storage capacity for lipids in SAT, AT accumulates intra-abdominally in visceral adipose tissue (VAT) and in other ectopic depots, such as PVAT and EAT (Hammarstedt et al., 2018). VAT underlies obesity-associated inflammation and is highly detrimental to cardiometabolic health (Berg and Scherer, 2005; Chartrand et al., 2022; Neeland et al., 2018). Pre-adipocytes from VAT have a lower adipogenic capacity and greater macrophage infiltration than pre-adipocytes from SAT (Turer et al., 2012; Piche and Poirier, 2018; Neeland et al., 2019). VAT underlies obesity-associated cardiometabolic risk (Neeland et al., 2018). Notwithstanding the equal mass of PVAT and VAT and the proximity of EAT to the RV/LV myocardium, the role of ectopic AT depots in obesity-associated cardiometabolic risk must be evaluated within the framework of obesity. Ectopic AT depots such as PVAT and EAT exert their actions in the context of excessive adiposity, particularly of VAT expansion and the associated systemic low-grade inflammation (Rana and Neeland, 2022; Ramo et al., 2024).

#### Epicardial adipose tissue

EAT mostly resides in the atrioventricular and interventricular grooves, where it wraps around the coronary arteries (Piche and Poirier, 2018). In the interventricular groove, EAT wraps around the LAD coronary artery, and the absence of any structure between EAT and the LAD artery adventitia facilitates bidirectional exchanges between AT and the arterial layers (Sacks and Fain, 2007). The release of 4-hydroxynonenal (HNE) from the arterial walls induces the expression of adiponectin in healthy PVAT that promotes tetrahydrobiopterin (BH4)-mediated endothelial nitric oxide synthase function and may affect the redox state in human vasculature (Margaritis et al., 2013). Acting similarly to PVAT, EAT affects the vascular reactivity and pathology of the LAD coronary artery (Badimon et al., 2024). Perivascular adipocytes from inflamed EAT contribute to adventitial inflammatory cell recruitment and intimal inflammation through direct communication and vasocrine effects via the vasa vasorum, microvessels that pass through the adventitia (Kim et al., 2020). Furthermore, steady expansion of the EAT functionally alters the LAD coronary artery in the absence of CAD, similarly to how obesity alters the effects of PVAT on vascular function (Xia and Li, 2017; Song et al., 2025).

### Inflammation drives atherosclerosis

EAT thickness or volume correlates with high-sensitivity C reactive protein (hs CRP) levels in patients with COVID-19, diabetic peripheral arterial disease (PAD), and suspected metabolic syndrome (Emekli et al., 2022; Gong and Peng, 2021; Cho et al., 2018). Whether EAT thickness predicts myocardial injury in patients with COVID-19 is uncertain (Su et al., 2024; Kung et al., 2025). Importantly, correlations between epicardial thickness or volume and hs-CRP levels were not adjusted for the amount of visceral adipose tissue area (Luo et al., 2025; Fukushima et al., 2024).

Furthermore, atherosclerosis is nowadays recognized as a subacute inflammatory condition of the arterial wall (Soehnlein and Libby, 2021). Traditionally, the activation, injury, and dysfunction of endothelial cells (ECs) trigger atherosclerosis-mediated vascular inflammation through an inside-to-outside scheme (Libby, 2024). In turn, the inflamed arterial walls release pro-inflammatory cytokines such as tumor necrosis factor alpha (TNF-α), interleukin (IL)-6, and interferon, which may promote local inflammation in adjoining EAT (Fan et al., 2023). Data concerning PVAT and inflammatory markers were collected from EAT biopsies at the time of coronary artery bypass surgery (CABG) (Jolfayi et al., 2025; Cheng et al., 2008; Baker et al., 2006). Metabolic risk markers and pro-inflammatory agents, including resistin, tumor necrosis factor (TNF)-α, and angiotensinogen (AGT), were similarly expressed in EAT from coronary artery disease (CAD) patients and in omental AT from non-CAD subjects (Baker et al., 2006). However, the lack of clinical information at the time of CABG surgery and the unreported sites of EAT biopsies relative to coronary arteries hinder the interpretation of data. The absence of clinical context at the time of CABG surgery and the unspecified biopsy sites hinder meaningful interpretation of these data. Furthermore, PVAT resistin concentration was 17.12 ng g^-1^ at the time of CABG surgery in never-smokers and 51.9 ng g^-1^ in ever-smokers, while plasma interleukin (IL)-6 values were 3.64 pg mL^-1^ and 7.1 pg mL^-1^, respectively (Rachwalik et al., 2024).

Furthermore, the CD11c/CD206 concentration ratio was three times greater in macrophages from the right ventricular EAT than in macrophages from atrioventricular EAT at the time of CABG surgery (Jolfayi et al., 2025).

Conversely, in the outside-to-inside scheme, EAT inflammation may spread to the adventitia and exacerbate atherosclerotic alterations in the intima and ECs (Ahmadieh et al., 2020; Hara and Sata, 2024). Human perivascular—and particularly pericoronary pre-adipocytes—produce more pro-inflammatory cytokines than pre-adipocytes from other *ectopic* depots. Proliferative vasa vasorum plays an important role in the progression of EAT-related atherosclerotic vascular alterations (Kim et al., 2020). PCAT inflammation leads to the development and destabilization of coronary atherosclerotic plaques (Mazurek and Opolski, 2015). Independent of the EAT volume and BMI, EAT attenuation is associated with high coronary calcium scores in men at a high risk for cardiovascular disease (Franssens et al., 2017).

#### Pericoronary adipose tissue attenuation

Coronary computerized tomography angiography (CCTA) enables the evaluation of localized PVAT inflammation (Antonopoulos et al., 2017). PVAT inflammation induces a gradient from the lipid-rich and less aqueous phase close to healthy arteries to a lipid-poor and more aqueous phase close to inflamed arteries (Oikonomou et al., 2019). The inflammation-related gradient results in CT attenuation ranging from more negative to less negative values (−190 to − 30 Hounsfield units [HU]) (Oikonomou et al., 2019; Oikonomou et al., 2018). PCATa is evaluated in 20 concentric, cylindrical, 1-mm-thick layers from the outer adventitia, extending from one to five centimeters from the coronary artery ostium (Antonopoulos et al., 2017). The average attenuation of AT over the region of interest defines the attenuation index (Antonopoulos et al., 2017). The PCATa index is greater than EAT density and serves as a convenient, noninvasive marker of coronary inflammation, independent of the presence or absence of coronary plaque (Bao et al., 2022). In addition to inflammation, increased fibrosis and vascular reactivity may modify PCAT attenuation (Grodecki et al., 2025). The reliable assessment of PCATa depends on numerous technical and methodological factors. They include consistent tissue imaging, site measurements, and scanner parameters such as HU threshold and tube voltage, along with artificial intelligence-based algorithms (Antonopoulos et al., 2023). All these factors need to be standardized before clinicians fully accept the usefulness of PCATa as a diagnostic and prognostic tool in the management of CAD (Oikonomou et al., 2021; Tan et al., 2023; Sagris et al., 2022; Grodecki et al., 2025; Oikonomou et al., 2019).

PCATa aims to detect atherosclerotic plaques with a high inflammatory burden and reduced collagen synthesis, which are prone to rupture or erosion and are therefore associated with major adverse cardiovascular events (Grodecki et al., 2025; Antonopoulos et al., 2017). Innate immunity, characterized by cytokine release from macrophages, and adaptive immunity, involving the recruitment of T lymphocytes, activated T-helper 1 lymphocytes, and production of interferon (IFN), underlie the progression of coronary artery atherosclerosis (Libby, 2021). The proximity of PCAT to the adventitia and the key role of inflammation in plaque vulnerability underlie the strong interest in PCTAa (Oikonomou et al., 2019). The PVATa index allows detection and monitoring of highly inflamed atherosclerotic plaques in the coronary arteries with the aim of predicting the risk of major adverse cardiovascular events (Antonopoulos et al., 2017). However, the association between PCATa and atherosclerotic plaque inflammation adds modest predictive discrimination when combined with standard cardiovascular risk scores (Grodecki et al., 2025). Whether CT PCATa is a reliable clinical marker of impending coronary events remains to be confirmed (Lin et al., 2021; Wen et al., 2023; Sagris et al., 2022; Fan et al., 2023; Guglielmo et al., 2024; Nerlekar et al., 2020).

In addition to the prediction of impending coronary events, PCATa allows following the progression of non-calcified coronary plaque for over 12 months in type-2 diabetic patients and correlates it with clinical outcomes after adjustments for clinical factors and CT angiography findings in patients with non-obstructive CAD (Overgaard et al., 2025; Zheng et al., 2025; Biradar et al., 2025). Hence, PCATa may serve as a marker of subclinical atherosclerotic progression.

### PCTAa and systemic adiposity

**FIGURE 1:**
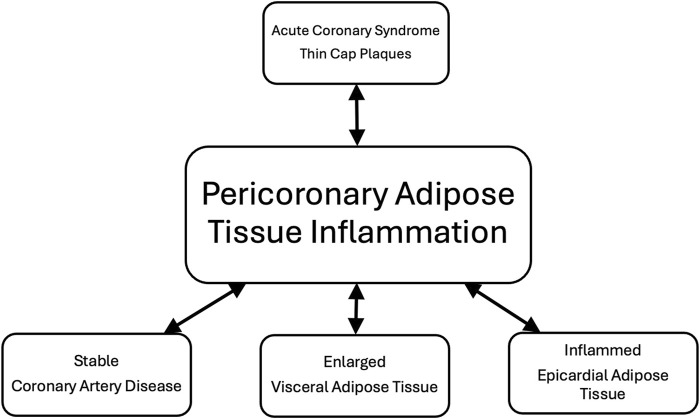
Coronary artery disease, visceral adipose tissue, and epicardial adipose tissue contribute to PCAT inflammation. Acute coronary syndrome (ACS)-associated inflammatory, thin-cap fibroatheroma, and macrophage-rich coronary plaque increase PCAT inflammation in men (Kinoshita et al., 2024). However, it is unclear whether longstanding VAT, inflamed EAT, and stable CAD can increase PCAT inflammation to the same extent as ACS. Preliminary data indicate that PCAT inflammation may overlap in ACS and stable CAD (Lin et al., 2021).

A close association between atherosclerotic vascular inflammation and PCATa is based on an overriding inside-to-outside scheme and EAT inflammatory state (Hara and Sata, 2025). The continued nutrient intake and energy expenditure mismatch in patients with untreated obesity exceed the capacity of the subcutaneous AT to store lipids, leading to the accumulation and inflammation of visceral and ectopic AT in the liver (hepatic steatosis), the kidneys (renal sinus fat), the pancreas, and around the heart (EAT) (Hammarstedt et al., 2018). Enlargement and inflammation of VAT promotes local and systemic inflammation, contributing to the atherosclerotic process (Powell-Wiley et al., 2021; Rana and Neeland, 2022). Furthermore, an enlarged and inflamed EAT because of type-2 diabetes or heart failure with preserved ejection fraction hastens the development and progression of coronary artery atherosclerosis through an outside-to-inside scheme (Manubolu et al., 2024; Hara and Sata, 2025; Song et al., 2025). Hence, vascular atherosclerotic inflammation is not the only cause of PCATa in patients with obesity. Depending on the relative contribution of VAT and EAT to vascular inflammation, PCATa may overestimate or underestimate coronary atherosclerotic plaque inflammation (Figure 1).

### Obesity, global longitudinal strain, and left ventricular subclinical systolic dysfunction

Various imaging modalities were investigated in the setting of obesity and PVAT. LV GLS was significantly lower in 589 patients with class I–III obesity than in 100 patients with normal body weight, although LV GLS remained above the normal value (20%) even in patients with class-III obesity (Huang et al., 2023). Notably, while LV GLS is a robust and reproducible echocardiographic parameter, foreshortening of the two-dimensional (D) LV apical views may lead to the overestimation of LV GLS (Smiseth et al., 2025). LV GLS is inversely related to BMI in patients with BMI ranging from normal (<25) to class-III obesity (≥30) (Prajapati et al., 2025). The decline in LV GLS in patients with elevated BMI is attributed to obesity-mediated systemic inflammation (Huang et al., 2023). Three-D tissue-tracking of LV myocardial strain by cardiac magnetic resonance imaging (MRI) corroborated the lower LV peak GLS of patients with insulin resistance and a mean BMI of 29.8 compared to that of healthy controls with a mean BMI of 21.2 (Liu et al., 2022). Finally, 6 months after metabolic bariatric surgery (MBS), LV GLS increased from 16.3 at baseline to 18.2, while BMI decreased from 48.4 at baseline to 35.4 in 38 patients with a mean age of 41 years (Piche et al., 2021).

Healthy subjects and diabetic patients with overweight or obesity have lower LV GLS than their normal-weight counterparts (Blomstrand et al., 2018). However, LV GLS is similar in healthy subjects who are overweight and obese, while LV GLS is lower in diabetic patients with obesity than in diabetic patients who are overweight (Blomstrand et al., 2018). LV GLS by 3D-echocardiography was associated with the VAT mass index determined by electric bioimpedance analysis in 195 diabetic patients with a BMI of 28.6 (Martinez-Dominguez et al., 2025). When measured by cardiac MRI, biventricular strain and strain were independently associated with EAT volume in 69 diabetic patients (Zhu et al., 2023). Finally, in 589 patients with an average BMI of 37.5, VAT measured by MRI was independently associated with the LV GLS peak (β = −2.684 and *p* = 0.016) (Huang et al., 2023).

### Epicardial adipose tissue and left ventricular global longitudinal strain

EAT thickness is commonly estimated by echocardiography. In 192 patients with BMI ≥35 without cardiovascular diseases, LV GLS and EAT thickness were inversely related, as evidenced by LV GLS of −17.6%, −17.1%, and −16.3% for EAT thickness of <3.8, 3.8–5.4, and >5.4 mm, respectively, with a β coefficient of −0.329 and *p* = 0.019 (Chin et al., 2023). When other significant univariable determinants of 3D-LV GLS are entered into the multiple linear regression model, EAT volume is an independent determinant of 3D LV GLS with a standardized β coefficient of 0.512 and *p* < 0.001 (Ng et al., 2016). Similarly, multivariate linear regression analysis indicates that EAT thickness measured by 2D-echocardiography is independently associated with LV GLS in type-2 diabetic patients (Song et al., 2022). One study of 71 subjects at risk for heart failure reported no association between regional EAT volume (anterior, lateral, and inferior regions) and LV GLS (Hearon et al., 2023). However, all the subjects had a mean EAT volume within the normal range for healthy subjects (Shmilovich et al., 2011).

### Epicardial adipose tissue and myocardial blood flow

EAT thickness independently predicts coronary microvascular disease (CMD) and coronary blood flow reserve (CFR) in patients with metabolic syndrome (Tok et al., 2013). Not unexpectedly, EAT thickness inversely correlates with CFR in women with chest pain and normal epicardial coronary arteries (Sade et al., 2009). However, only the EAT volume index and not the total EAT volume is the independent predictor of CMD (Abusnina et al., 2025). EAT accumulation worsens LV diastolic function, and increasing EAT index correlates with the onset of heart failure with preserved ejection fraction in patients with coronary artery disease (Nakanishi et al., 2017; Mahabadi et al., 2022).

Importantly, enlarging the EAT volume independently reduces LV GLS without affecting the global radial or circumferential strains in patients with preserved LV ejection fraction and patent coronary arteries (Maimaituxun et al., 2020). The underlying mechanisms that link the EAT volume to LV GLS remain unclear. The release of cytokines by an enlarged and inflamed EAT may lower regional myocardial contractile function through paracrine and vasocrine effects (Maimaituxun et al., 2020). Quantitative non-invasive assessment of coronary microvascular function with stress perfusion MRI revealed abnormal LV sub-endocardial perfusion in women with obesity and normal epicardial coronary arteries (Markley et al., 2023). In summary, EAT accumulation may reduce regional LV systolic function through cytokine-mediated coronary microvascular dysfunction, endangering LV sub-endocardial perfusion.

#### Epicardial adipose tissue and cardiac steatosis

Myocardial (intramyocellular) lipid contributes to the development of cardiovascular disease in patients with obesity (McGavock et al., 2006). Hence, myocardial accumulation of triglycerides, e.g., cardiac steatosis, may account for the decrease in LV GLS in patients with obesity and enlarged EAT. MBS leads to a large loss of weight, VAT, and EAT volume or thickness without any changes in the myocardial triglyceride content (MTGC) measured by ^1^H-magnetic resonance (MR) spectroscopy. Three and six months after MBS, MTGC did not change in 12 patients with an average BMI of 43.9at baseline (Mikhalkova et al., 2018). Six months after MBS, VAT and EAT decreased by 47% and 27%, respectively, without any changes in MTGC in 70 patients, and VAT and EAT decreased by 35% and 7%, respectively, without any MTGC changes in 10 patients (Gaborit et al., 2012; van Schinkel et al., 2014). Six months after MBS, MTGC did not change in 28 type-2 diabetic patients with a baseline average BMI of 42.6 and in 18 patients (without type-2 diabetes) with a baseline average BMI of 41.0 (Hannukainen et al., 2018). However, 32 months after MBS, VAT decreased by 46%, EAT decreased by 33%, and MTGC decreased by 40% in 21 patients with an average BMI of 43.2 at the baseline (Abdesselam et al., 2016). In a meta-analysis of the changes in EAT after MBS, only one study reported a non-significant EAT reduction among 24 studies (Pereira et al., 2023). In summary, the only consistent changes in body composition within 6 months of MBS are a decrease in VAT and EAT. Hence, while cardiac steatosis is associated with VAT, it does not account for the improvement in LV GLS and, thereby, the subclinical LV function in the months that follow MBS.

## Conclusion

The perivascular adipose tissue surrounding coronary arteries plays a role in coronary plaque formation and the advancement of atherosclerosis through an outside-to-inside scheme, where pericoronary adipocytes produce more inflammatory cytokines than adipocytes from other *ectopic* adipose tissue depots. Importantly, from a vascular health perspective, non-invasive evaluation of pericoronary adipose tissue inflammation may help predict the risk of future ischemic events in patients with coronary artery disease and demonstrate the effectiveness of anti-inflammatory therapeutic approaches that aim to limit the burden of atherosclerosis. Finally, the effects of perivascular adipose tissue on vascular reactivity may unravel the links between thickened, enlarged epicardial adipose tissue and subclinical left ventricular dysfunction.

## Data Availability

The original contributions presented in the study are included in the article/supplementary material; further inquiries can be directed to the corresponding author.
